# Comparison of a high-definition three-dimensional digital camera system with a conventional state-of-the-art operation microscope for microsurgical anastomoses

**DOI:** 10.1038/s41598-023-37530-1

**Published:** 2023-07-05

**Authors:** Andreas M. Fichter, Constantin T. Wolff, Alex Grabenhorst, Leonard H. Koss, Achim von Bomhard, Markus Nieberler, Klaus-Dietrich Wolff, Lucas M. Ritschl

**Affiliations:** 1 Department of Oral and Maxillofacial Surgery, School of Medicine, Technical University of Munich, Klinikum Rechts Der Isar, Ismaninger Straße 22, 81675 Munich, Germany; 2 Department of Oral and Maxillofacial Surgery, University of Cologne, Kerpener Straße 62, 50937 Cologne, Germany

**Keywords:** Microendoscopy, Medical research, Imaging

## Abstract

Since its clinical implementation, microvascular surgery has depended on the continuous improvement of magnification tools. One of the more recent developments is a high-definition three-dimensional (3D) digital system (exoscope), which provides an alternative to the state-of-the-art operating microscopes. This study aimed to evaluate the advantages and disadvantages of this technology and compare it with its predecessor. The study included 14 surgeons with varying levels of experience, none of which had used a 3D optical system previously. Six of these surgeons performed five arterial and five venous anastomoses in the chicken thigh model with both the VITOM 3D exoscope-guided system and the Pentero operating microscope. These anastomoses were then evaluated for their quality and anastomosis time. The participants and the other eight surgeons, who had used the digital 3D camera system for microsurgical training exercises and vascular sutures, answered a questionnaire. The anastomosis time and number of complications were lower with the conventional microscope. Participants rated the image quality with the conventional microscope as higher, whereas the field of view and ergonomics were favorable in the digital 3D camera system. Exoscopes are optics suitable for performing simple microvascular procedures and are superior to classical microscopes ergonomically. Thus far, they are inferior to classical microscopes in terms of image quality and 3D imaging.

## Introduction

Microsurgery, especially microvascular surgery, has been implemented in a broad spectrum of surgical disciplines. The precondition for its application is always the improvement and diversification of the required materials and instruments. Specifically, the development of suitable magnification tools is necessary to operate on small structures precisely. The first records of the discovery and understanding of magnification date back to centuries before Christ and are linked to names such as Archimedes, Ptolemaeus, or Seneca, describing the phenomena of the burning glass, magnifying, or refraction features of water^1^ With increasing knowledge and technical equipment over thousands of years, compound microscopes were first constructed by Dutch spectacle makers Jansen and Lippershey around 1590 and shortly after augmented by adding a third lens^1–4^ This so-called Microscopium was improved by Hooke and Campani in the seventeenth century and, among other things, was used for the examination of wounds and scars^1,2,5–7^ With the desire and need for technical improvement and downsizing these bulky instruments, further innovations were made throughout the next centuries. Leading characters in the development of a modern microscope were Zeiss, Abbe, and Leitz. The close affiliation between technical and physical sciences, together with manufacturing skills, led to the design and development of modern microscopes in the late nineteenth century, being manufactured in the so-called Carl Zeiss Werke in Oberkochen, Germany^8,9^ The first recorded application of a monocular microscope for operative procedures in humans was done by the ear, nose, and throat specialist Nylén in 1921, which was shortly after modified by his head of department Holmgren by attaching a binocular Zeiss microscope^1,10,11^ Other disciplines, such as ophthalmology and plastic and reconstructive surgery, followed and adopted the operating microscope. Since then, further adjustments and modifications were made, improving the magnification and facilitating the utilization of these microscopes. This desire for even more refinements continues until the present day. Specifically, developments in digital image processing allow unimagined application possibilities in the field of microsurgery.

A more recent development is a three-dimensional (3D) camera system that has been adopted in multiple aspects of daily and professional lives. This high-definition visually guided 3D system has cameras outside the body at a distance of 25–75 cm from the surgical site. Compared with a modern surgical microscope, this digital 3D camera system is characterized by a smaller and more convenient device and allows ergonomically working with the help of 3D glasses.

In addition to magnification, another big issue in (super-) microsurgery has now been solved, i.e., physiological tremors. In vascular and lymphogen anastomoses of the lumina with a diameter of < 1.0 mm, eliminating physiological tremors facilitates the performance of this demanding procedure. Most recently, robot-assisted microsurgery has evolved as the final missing puzzle piece in this highly specialized field^12,13^ Modern robotic systems such as the “Symani surgical system” do not have optics but are compatible with both classic microscopes and exoscopes. The concept of robot-assisted surgery in the field of (super-) microsurgery depends on excellent magnification and image quality, which should ideally be based on digital data that can be transferred to remote screens/goggles to take full advantage of the remote robotic setup. Especially, for interventions in body cavities that are difficult or impossible to access by conventional means (skull base, pharynx, etc.), exoscope-based optics would be the ideal partner for robots. However, reservations remain regarding the exoscope’s image quality and 3D perception compared with the conventional binocular microscope.

**Figure 1:**
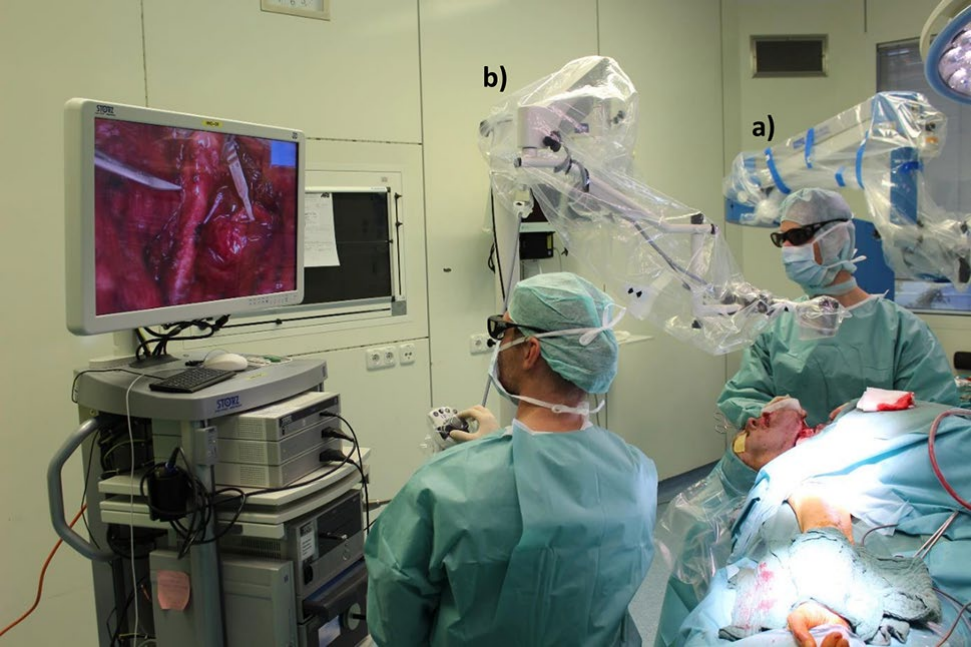
Technologies implemented to visualize the surgical target in microsurgery: (**a**) The surgical microscope as a conventional state-of-the-art operation microscope for microsurgical anastomoses, (**b**) The exoscope-guided system as a high-definition visually guided 3D system with cameras outside the body at a distance of 25–75 cm from the surgical site, allowing working conveniently and ergonomically with the help of 3D glasses and a monitor.

This study aimed to evaluate the possible advantages and disadvantages of an exoscope-guided 3D system in comparison with a state-of-the-art microscope in an in vitro setting and consider the results in the context of the emerging robotic era in microsurgery (see Fig. 1).

## Material and methods

This study included 14 surgeons with varying levels of experience, and none of them had any previous experience with exoscope-guided microsurgery. Six of them (two advanced and four highly experienced microsurgeons), who had previously used a standard operating microscope in both research-related and clinical practice, followed a standardized surgical procedure, performing a total of 20 end-to-end anastomoses each, which were then objectively evaluated. The other eight surgeons were considered as microsurgery trainees. They were participants of a 3-day microsurgery training course or were members of the department of Oral and Maxillofacial Surgery of the Technical University of Munich, Klinikum rechts der Isar, with basic microsurgical experience levels. They were given the opportunity to perform microsurgical training exercises and vascular sutures and answered the same questionnaire given to the former six surgeons.

Each participant received instructions regarding the general properties and various components of the digital 3D camera system, ensuring that they knew how to operate it.

### Surgical procedure and analysis

**Figure 2:**
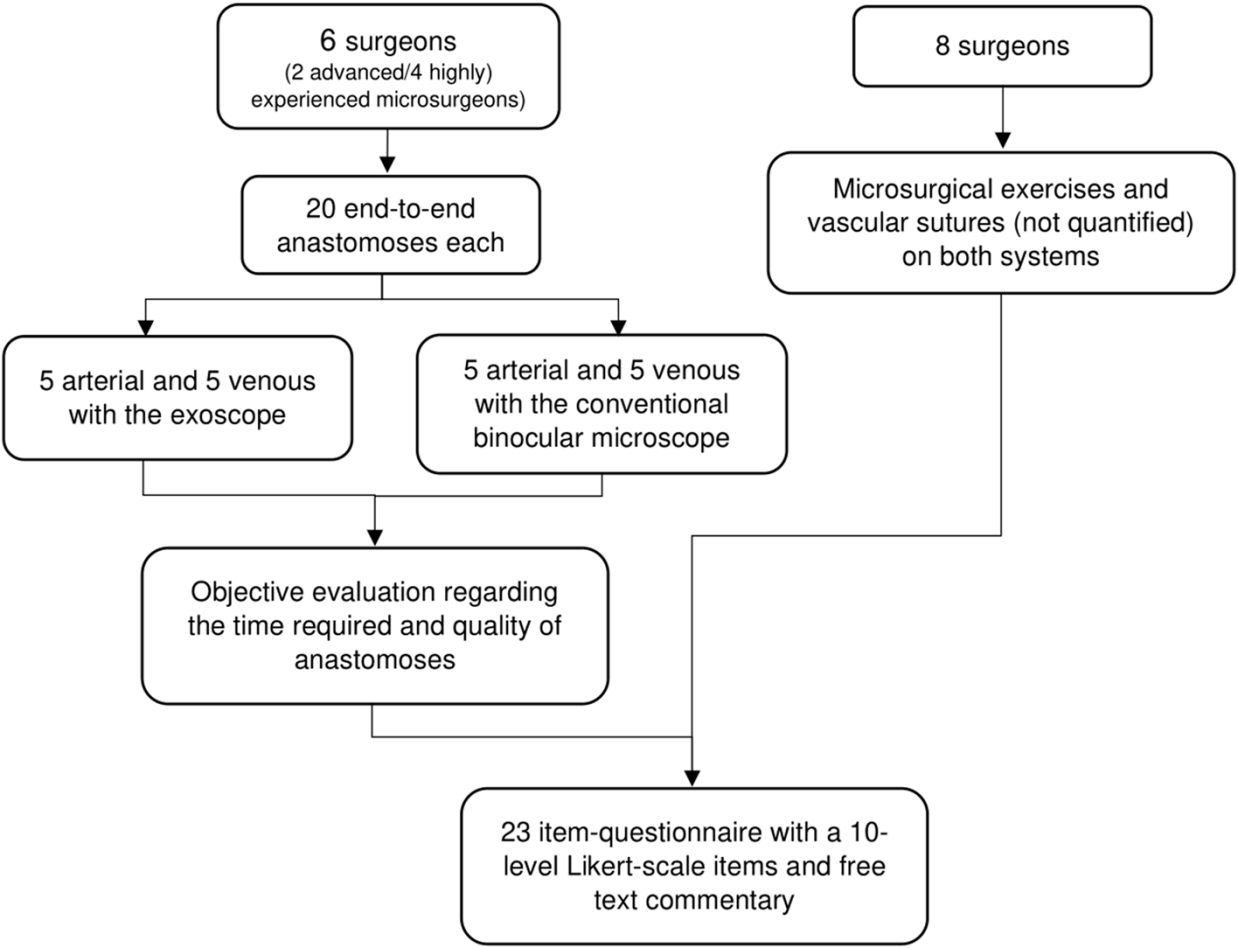
Setup of the study.

Surgical procedures were performed and evaluated according to a standardized protocol (see Fig. 2) and according to the legal national legislation without the necessity to obtain an approval by the institutional ethics committee of the Technical University of Munich, Klinikum rechts der Isar. Six participants performed 10 arterial and 10 venous anastomoses with the operating microscope type OPMI® Pentero® (served as the reference; INFRARED 800; Carl Zeiss Meditec AG; Oberkochen, Germany) and the exoscope-guided 3D system (Karl Storz GmbH; Tuttlingen, Germany) in the chicken thigh model using a 10–0 Ethilon suture (10–0 Ethilon®, Ethicon; Norderstedt, Germany)^14^ All participants used the same microsurgical instruments and equipment required for anastomosis (S&T AG; Neuhausen, Switzerland). Refrigerated chicken thighs (EDEKA Bio WWF Chicken Thigh 1 kg) were obtained in a nearby supermarket and disposed in an appropriate container after successfully completing the surgical procedures and evaluations.

**Figure 3:**
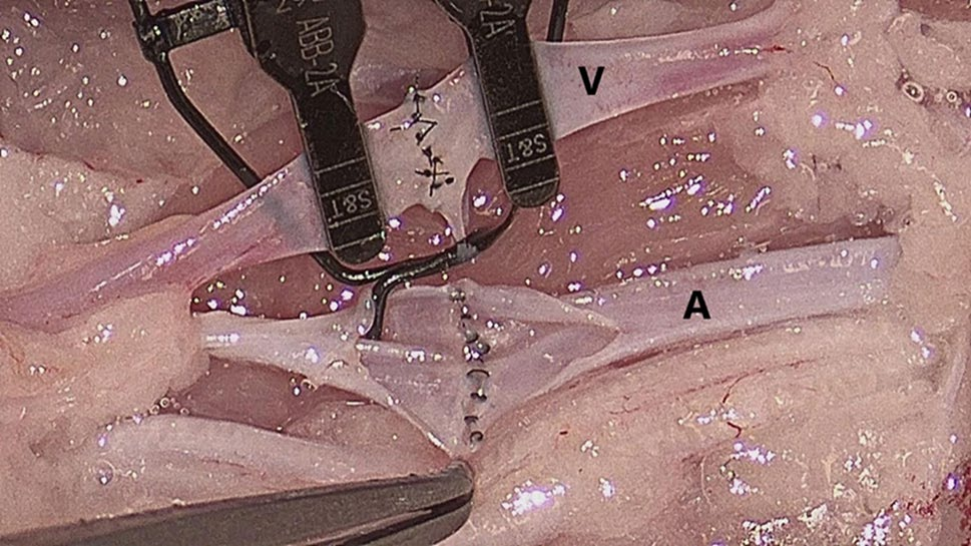
Anastomoses of the artery (**A**) and vein (**V**) as performed by the participants in the chicken thigh model. The artery has been opened to evaluate the quality of the sutures and its patency.

The time in minutes per arterial and venous anastomoses and the number of sutures performed were registered, and the time per suture in minutes was calculated. Each of the anastomoses was then randomized and evaluated independently by a blinded microsurgeon based on its patency and whether the back wall had been sewn on and the adventitia had been sewn in (see Fig. 3). The patency of the anastomoses was directly related to whether the posterior wall was sewed on and therefore only recognized when this was not the case.

### Questionnaire

Each of the participants was asked to describe their experience with an operating microscope, and their opinions on the two systems were evaluated with a questionnaire consisting of 10-level Likert scale items (1 = does not apply at all, 10 = fully applies) regarding handling, vision, ergonomics, magnification, and practicability (Table 1).

**Table 1:** Questionnaire.

Questions
On a scale from 1 to 10, how experienced are you in the handling/operation of an operating microscope?
The first use of the digital camera 3D system was a challenge for me
Overall handling the operating microscope/digital camera 3D system was very comfortable and easy
Handling the digital camera 3D system came more naturally and easier to me in comparison with the use of an operating microscope
The field of view was acceptable when using the operating microscope/digital camera 3D system
The image quality of the operating microscope and digital camera 3D system is comparable, even at higher zoom levels
On a scale from 1 to 10, how would you describe the contrast/sharpness/definition of the image quality when using the operating microscope/digital camera 3D system?
On a scale from 1 to 10, how would you describe the ergonomics/the overall comfort (sitting position, freedom of head movement, and overview) when using the operating microscope/digital camera 3D system?
It was easy for me to discern between the different tissue layers when using the operating microscope/digital camera 3D system
Being accustomed to the use of the digital camera 3D system and the different optics (monitor instead of a microscope) after having gained some experience with an operating microscope was surprisingly easy
The 3D image of the digital camera 3D system is comparable to the real-life situation and allows handling of the instruments in 3D space
Especially when using a higher zoom level, a noticeable camera lag was disturbing when using the digital camera 3D system
With higher zoom, image quality decreased when using the digital camera 3D system
The achievable level of zoom was high enough even for nerve reconstruction/small-vessel anastomosis when using the digital camera 3D system
In my opinion, the digital camera 3D system can be used to perform microsurgical techniques (micro dissection/micro anastomosis)
The eye–hand/hand–instrument coordination was problematic when using the operating microscope/digital camera 3D system
When using the digital camera 3D system, I felt distracted because I could see both the operation field and the surrounding operating theater (unlike looking through a tunnel when using a microscope.)

Participants were also asked to describe any advantages that either of the two systems had over the other in free text form and identify any problems they encountered when using the digital camera 3D system.

From all participants a written informed consent was obtained before carrying out the questionnaire.

### Statistical analyses

Data were analyzed with IBM SPSS Statistics for Windows version 23.0 (IBM Corp., Armonk, NY, USA). Figures were generated with Excel® (Microsoft Excel® 14.2.3 for Mac, Microsoft Corp., Redmond, WA, USA). The Wilcoxon test was used to test the hypothesis between anastomoses performed with the operating microscope type OPMI® Pentero® and VITOM® 3D exoscope and participants’ answers regarding those questions that stood in a direct comparison of the two systems. All statistical hypothesis testing was performed on exploratory two-sided 5% significance levels. To identify any tendencies among participants’ answers, a qualitative evaluation was performed on the rest of the questionnaire.

### Accordance statement

All methods were carried out in accordance with relevant guidelines and regulations.

## Results

### Surgical results

The median duration by each of the six participants for anastomosis and per suture along with their corresponding range and the incidence of back wall catch or intraluminal adventitia with each modality are displayed in Table 2.

**Table 2:** Median time needed by each participant, time needed per suture, and number of times the back wall was sewed on and the adventitia sewn in. A = artery, V = vein.

Participant	1		2		3		4		5		6	
Vessel	A	V	A	V	A	V	A	V	A	V	A	V
Digital 3D camera system												
Median time in [min]	25.0 (20.9–25.4)	31.3 (30.7–37.2)	30.2 (25.0–35.7)	35.3 (30.5–40.3)	8.3 (7.3–10.4)	9.8 (9.2–10.4)	10.1 (9.8–12.2)	12.7 (9.8–12.8)	10.3 (8.9–11.5)	12.9 (10.8–13.7)	9.3 (8.1–11.2)	14.5 (11.5–15.0)
Median time per suture in [min]	2.3 (1.9–2.5)	2.6 (2.6–3.1)	3.0 (2.8–3.9)	2.9 (2.2–3.6)	1.2 (1.1–1.5)	1.2 (1.1–1.3)	1.1 (1.1–1.4)	1.3 (1.1–1.4)	1.2 (0.9–1.3)	1.5 (1.2–1.6)	1.2 (1.0–1.6)	1.6 (1.2–1.8)
Back wall was sewed on	0	3	1	2	0	1	0	0	0	1	0	2
Adventitia sewn in	3	1	1	1	0	0	0	4	1	0	2	3
Operating microscope												
Median time in [min]	21.6 (20.4–22.8)	28.9 (27.9–30.9)	27.0 (24.6–33.8)	34.2 (31.4–40.1)	6.8 (6.5–7.3)	6.8 (6.3–8.5)	8.7 (8.0–9.8)	9.1 (8.4–9.9)	7.4 (6.9–9.2)	9.1 (1.9–10.4)	9.7 (8.7–10.2)	14.3 (11.0–18.2)
Median time per suture in [min]	1.9 (1.9–2.1)	2.5 (2.4–2.6)	2.9 (2.5–3.8)	2.9 (2.7–3.7)	0.9 (0.9–1.2)	1.1 (0.9–1.2)	0.9 (0.9–1.2)	0.9 (0.9–1.1)	0.9 (0.8–1.0)	1.0 (0.9–1.2)	1.3 (1.2–1.5)	1.4 (1.2–2.0)
Back wall was sewed on	0	1	0	2	0	0	0	0	0	0	0	0
Adventitia sewn in	1	1	1	0	0	2	0	0	0	2	1	3

**Figure 4:**
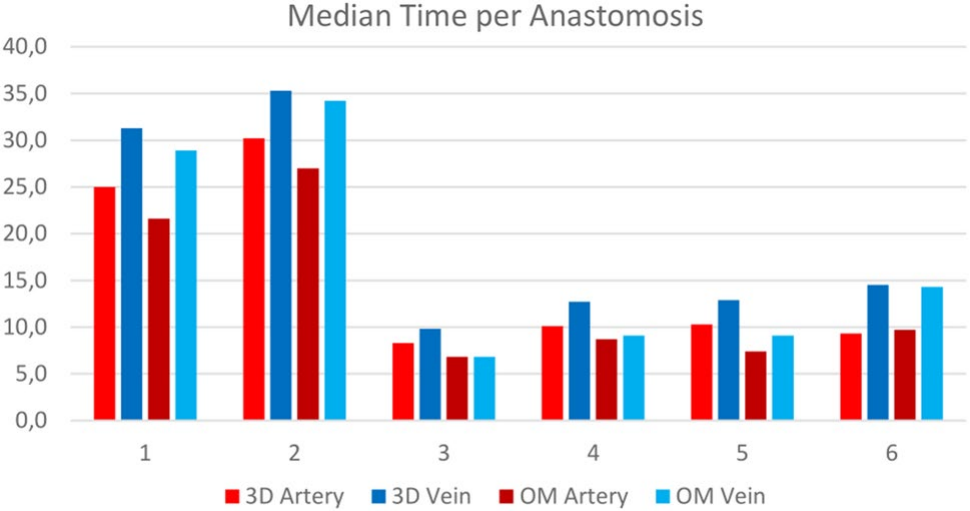
Median time [s] in minutes required by each of the six surgeons for their arterial and venous end-to-end anastomoses on the operating microscope (OM) and digital 3D camera system (3D). Numbers 1 and 2 represent surgeons with advanced, and numbers 3–6 surgeons with professional microsurgical skills.

The time required for anastomosis on the arterial vessel was higher with the digital camera 3D system (*med* = 10.4 min [8.9–29.4]) than with the operating microscope (*med* = 9.2 min [6.9–28.5]). According to the Wilcoxon test, a significant difference with a strong effect was found (*p* = 0.046, *r* = 0.81). Similarly, participants required more time on average for the venous anastomoses with the digital camera 3D system (*med* = 13.2 min [9.8–34.5]) than with the operating microscope (*med* = 12.0 min [7.1–34.9]). Unlike the arteries, no significant difference was found (*p* = 0.116, *r* = 0.64) (see Fig. 4).

Accordingly, participants required less time per knot when performing arterial anastomoses with the operating microscope (*med* = 1.2 min [0.9–3.1]) than with the digital camera 3D system (*med* = 1.3 min [1.2–3.2]), and a significant difference with a strong effect was observed (*p* = 0.046, *r* = 0.81). Regarding venous anastomoses, participants were also faster per suture when using the operating microscope (*med* = 1.3 min [0.9–3.1]) than when using the digital camera 3D system (*med* = 1.5 min [1.2–2.9]), but the difference was not significant (*p* = 0.173, *r* = 0.56).

Of all arterial anastomoses on the digital camera 3D system (*med* = 0.0 [0.0–1.0]), only one vessel had the posterior wall sewn on. With the operating microscope (*med* = 0.0 [0.0–0.0]), this was never the case. A significant difference was not found (*p* = 0.317, *r* = 0.41). For venous anastomoses, participants suture the posterior wall more often when using the digital camera 3D system (*med* = 1.5 [0.0–3.0]) than when using the operating microscope (*med* = 0.0 [0.0–2.0]). Again, the difference was not significant (*p* = 0.063, *r* = 0.76).

The adventitia of the arteries was sewn on more often when the digital camera 3D system was used (*med* = 1.0 [0.0–3.0]) than when the operating microscope was used (*med* = 0.5 [0.0–1.0]), and no significant difference was found (*p* = 0.102, *r* = 0.67). In venous anastomoses, no clear tendency regarding the superiority of one of the systems could be determined: operating microscope (*med* = 1.5 [0.0–3.0]) and digital camera 3D system (*med* = 1.0 [0.0–4.0]). No significant difference was found in this aspect (*p* = 1.00, *r* = 0.00).

### Questionnaire results

All 14 participants answered the 23-item questionnaire. The participants found the general handling more pleasant with the digital camera 3D system (*med* = 7.0 [2.0–9.0]) than with the operating microscope (*med* = 6.5 [1.0–9.0]), but no significant difference was found (*p* = 0.578, *r* = 0.15). The field of view of the digital camera 3D system (*med* = 8.0 [7.0–10.0]) was also rated better than that of the operating microscope (*med* = 8.0 [4.0–10.0]), and a significant difference was found (*p* = 0.063, *r* = 0.50).

All participants rated the image quality higher with the operating microscope (*med* = 9.0 [7.0–10.0]) than with the digital camera 3D system (*med* = 7.0 [3.0–8.0]). The difference proved to be significant, and the effect was strong (*p* = 0.001, *r* = 0.89).

Ergonomics were better with the digital camera 3D system (*med* = 9.0 [4.0–10.0]) than with the operating microscope (*med* = 6.5 [3.0–8.0]). This also showed a significant difference with a strong effect (*p* = 0.014, *r* = 0.66).

However, the participants found it easier to recognize different tissue layers on the operating microscope (*med* = 8.5 [7.0–10.0]) than on the digital camera 3D system (*med* = 7.5 [6.0–9.0]). This difference was also significant, and the effect was strong (*p* = 0.011, *r* = 0.68).

**Figure 5:**
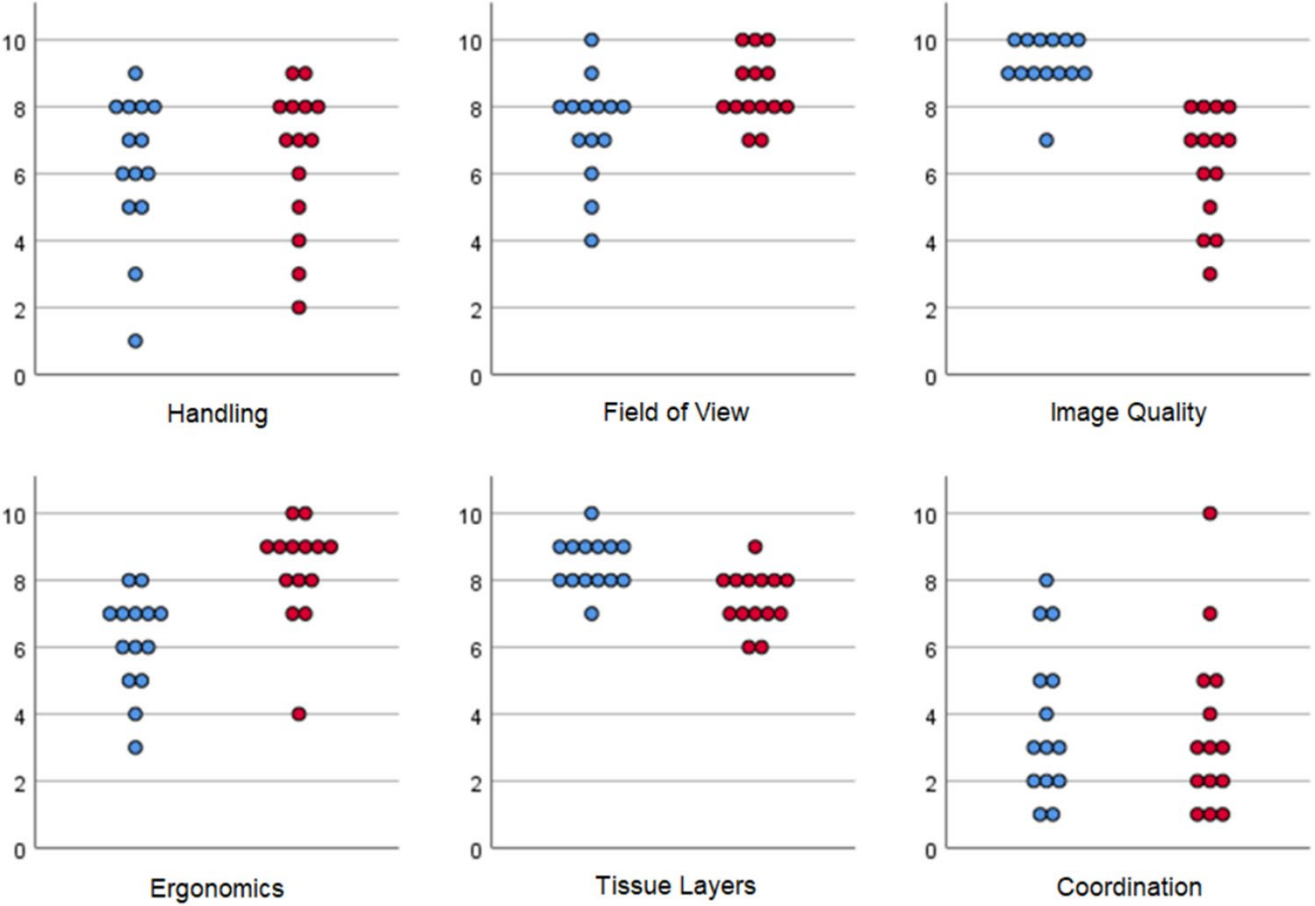
Distribution of participants’ answers to the questions directly comparing the two systems. red = digital 3D camera system, blue = operating microscope.

The eye–hand/eye–instrument coordination appeared to function without issue with both the operating microscope (*med* = 3.0 [1.0–8.0]) and the digital camera 3D system (*med* = 3.0 [1.0–10.0]). Accordingly, no significant difference was found (*p* = 0.478, *r* = 0.19) (see Fig. 5).

Among the participants, five had limited experience on using an operating microscope, three were moderately experienced, and the rest rated their experience as high or very high. The participants experienced mixed levels of difficulty the first-time they used the digital camera 3D system. However, many surgeons found the initial use of the exoscope easier and more natural than their first use of a standard operating microscope, especially after having gained some experience with the latter. Six of the participants did not find the image quality to be comparable between the two systems.

Although most of the participants noticed a decrease in the image quality and a marked camera lag when using higher zoom levels with the digital camera 3D system, all of them, except one, found the 3D image to be comparable to the real-life situation and the achievable zoom level to be adequate for small-vessel anastomosis or even nerve reconstruction. Accordingly, they also deemed it adequate for microsurgical procedures. While some of the surgeons struggled with the unfiltered surroundings during their use of the digital camera 3D system, others did not consider that as a distraction.

## Discussion

The evaluation of both objective surgical performance and subjective impressions aimed to identify the advantages and disadvantages of the 3D system compared with the standard operating microscope. The surgical results concerning the operating time required to perform an anastomosis were significantly shorter with the operating microscope than with the digital 3D camera system. Moreover, the complication rate when performing an anastomosis (i.e., back wall sutured on and adventitia was sewed in) was higher when using the digital 3D camera system, although not statistically significant. These results may indicate that the digital 3D camera system is inferior to the operating microscope in terms of valuable operating time and avoidable complications. However, the implementation of a new operating system may prolong the operating time and may aggravate the conduction of known procedures in first-time applications with consequent complications. This consideration is corroborated by the results of Sassu et al., who performed several anastomoses in a rat’s femoral artery and encountered a reduction in operating time of > 30% compared with the first-time application^15^.

The questionnaire survey of the study participants revealed different results (see Table 3). Image quality and the distinction of various tissue layers showed to be significantly superior in the operating microscope, especially at higher zoom levels, which becomes increasingly important with decreasing vascular diameter. At higher zoom levels, the image quality of the digital 3D camera system decreased noticeably, becoming blurred and started lagging.

**Table 3:** Results of the questions not analyzed with the Wilcoxon test. 1 = does not apply at all, 10 = fully applies.

Questions	Distribution of the questionnaire results on a 10-level Likert scale									
	1	2	3	4	5	6	7	8	9	10
On a scale from 1 to 10, how experienced are you in the handling/operation of an operating microscope?		28.6% (*n* = 4)	7.1% (*n* = 1)		7.1% (*n* = 1)	14.3% (*n* = 2)	7.1% (*n* = 1)	14.3% (*n* = 2)	14.3% (*n* = 2)	7.1% (*n* = 1)
The first use of the digital 3D camera system was a challenge for me	7.1% (*n* = 1)	7.1% (*n* = 1)	7.1% (*n* = 1)	7.1% (*n* = 1)	21.4% (*n* = 3)	7.1% (*n* = 1)	14.3% (*n* = 2)	14.3% (*n* = 2)		14.3% (*n* = 2)
Handling the digital 3D camera system came more naturally and easier to me in comparison with the use of an operating microscope	7.1% (*n* = 1)		7.1% (*n* = 1)	14.3% (*n* = 2)	7.1% (*n* = 1)		14.3% (*n* = 2)	21.4% (*n* = 3)	14.3% (*n* = 2)	14.3% (*n* = 2)
The image quality of the microscope and the digital 3D camera system is comparable, even at higher zoom levels	7.1% (*n* = 1)	21.4% (*n* = 3)	14.3% (*n* = 2)		14.3% (*n* = 2)	21.4% (*n* = 3)	14.3% (*n* = 2)	7.1% (*n* = 1)		
Being accustomed to the use of the 3D camera system and the different optics (monitor instead of a microscope) after having gained some experience with an operating microscope was surprisingly easy	7.1% (*n* = 1)		14.3% (*n* = 2)		7.1% (*n* = 1)		7.1% (*n* = 1)	14.3% (*n* = 2)	42.9% (*n* = 6)	7.1% (*n* = 1)
The 3D image of the 3D camera system is comparable with the real-life situation and allows handling of the instruments in the 3D space			7.1% (*n* = 1)			14.3% (*n* = 2)	21.4% (*n* = 3)	35.7% (*n* = 5)	21.4% (*n* = 3)	
Especially when using a higher zoom level, a noticeable camera lag was disturbing when using the 3D camera system	7.1% (*n* = 1)	7.1% (*n* = 1)	7.1% (*n* = 1)		21.4% (*n* = 3)		14.3% (*n* = 2)	21.4% (*n* = 3)	21.4% (*n* = 3)	
With higher zoom, the image quality decreased when using the 3D camera system		7.1% (*n* = 1)		7.1% (*n* = 1)			21.4% (*n* = 3)	28.6% (*n* = 4)	28.6% (*n* = 4)	7.1% (*n* = 1)
The achievable level of zoom was high enough even for nerve reconstruction/small-vessel anastomosis when using the 3D camera system			7.1% (*n* = 1)			21.4% (*n* = 3)	21.4% (*n* = 3)	21.4% (*n* = 3)		28.6% (*n* = 4)
In my opinion, the 3D camera system can be used to perform microsurgical techniques (micro dissection/micro anastomosis)						7.1% (*n* = 1)	35.7% (*n* = 5)	21.4% (*n* = 3)	14.3% (*n* = 2)	21.4% (*n* = 3)
When using the 3D camera system, I felt distracted because I could see both the operation field and the surrounding operating theater (unlike looking through a tunnel when using a microscope.)	14.3% (*n* = 2)	21.4% (*n* = 3)	7.1% (*n* = 1)	7.1% (*n* = 1)		14.3% (*n* = 2)	21.4% (*n* = 3)		7.1% (*n* = 1)	7.1% (*n* = 1)

Other studies have reported the same impression^16,17^ This might be caused by the direct transmission of visual impressions in the operating microscope without the help of a camera and its required monitoring screen. Unsurprisingly, the field of view and ergonomics were rated better in the digital 3D camera system. Multiple studies and reports of various specialties in different application setups have confirmed this observation^18–22^ This benefit could be further enhanced by combining the digital 3D camera system with additional technologies such as robotic microsurgical systems, the most recent advancement in microsurgical equipment^23^ Microsurgical procedures that prove to be particularly difficult because of vessel size, reachability, or other adverse circumstances using a standard approach and an operating microscope have been facilitated with the use of a digital 3D camera system implemented in an exoscope or (flexible) endoscope. Their combination also resembled another step in the history of robotic surgery generally and robotic telesurgery specifically^12^ Although the field of its application is still limited, separating the body of expertise, i.e., the surgeon, from the area of operation entirely is made possible by replacing a standard microscope with a digital 3D camera system.

Besides our statistical results, this study furthermore emphasizes the need for intense microsurgical training as a foundation, and with the help of which, such a high level of microsurgical proficiency can be achieved. For an objective assessment of the anastomoses, we evaluated their patency in a standardized manner, which is frequently considered an objective qualitative parameter used as a surrogate to describe anastomosis success^24,25^ Specifically, we examined whether the back wall of a vessel was sewed on and if the adventitia, normally surrounding the vessel circumferentially, was sewed inside the vessel lumen by mistake. The finding that the back wall was sewed on 13 times and the adventitia was sewn 27 times inside the vessel lumen (Table 2) of all 120 anastomoses performed during an experimental study under utmost convenient preconditions in comparison with ordinary conditions in the operating room shows the significance of efficient and constant microsurgical training. In this study, both systems, the operating microscope and the digital 3D camera system, were equipped with large screens serving as observation and demonstration tools for teaching students and younger surgeons and therefore accomplish the purpose of microsurgical education, as demonstrated before by De Virgilio^26^.

Nevertheless, this study has some limitations. The limited number of surgeons and their subjectively perceived impressions and opinions may not be permitted to produce definite conclusions to issue a clear recommendation for either of the two optical systems, although the surgical data suggest a certain advantage of the conventional optical microscope. Regardless, no inconsiderate transmission to an application for a microsurgical operation on a patient may be made because we investigated the use of a digital 3D camera system in an experimental setting outside the operating room and its special conditions, even though multiple studies have unveiled promising reports and results hereof^16,19,20,27,28^ Further research is necessary to evaluate the comparability between 3D systems and conventional microscopes in their clinical application, ideally involving a greater number of surgeons who have had experience with both systems leading up to the study.

## Conclusions

The tools used for microsurgery have undergone continuous development, and the digital 3D camera system will not be the last. However, technical improvements and prolonged implementation in practice appear to be necessary before it can take its place as a state-of-the-art piece of equipment in the operating room. With these adjustments, its ergonomic advantages, especially when paired with other technologies such as operating robots or implemented endoscopes, could justify an equally coexisting or at least partially a replacement of the standard surgical microscope.

## Data Availability

All data generated or analyzed during this study are included in this published article.
